# First report of highly pathogenic *Echinococcus granulosus* genotype G1 in dogs in a European urban environment

**DOI:** 10.1186/s13071-015-0796-3

**Published:** 2015-03-26

**Authors:** Leidi Laurimaa, John Davison, Karmen Süld, Liivi Plumer, Ragne Oja, Epp Moks, Marju Keis, Maris Hindrikson, Liina Kinkar, Teivi Laurimäe, Jaana Abner, Jaanus Remm, Peeter Anijalg, Urmas Saarma

**Affiliations:** Department of Zoology, Institute of Ecology and Earth Sciences, University of Tartu, Vanemuise 46, 51014 Tartu, Estonia

**Keywords:** Dog, Echinococcosis, Emerging infectious diseases, Molecular diagnostics, Noninvasive genetics, Parasites, Urban dogs, Zoonoses

## Abstract

**Background:**

*Echinococcus granulosus* and *E. multilocularis* are tapeworm parasites of major medical and veterinary importance, causing cystic and alveolar echinococcosis, respectively. Both diseases are listed among the most severe parasitic diseases in humans, representing 2 of the 17 neglected diseases prioritised by the World Health Organisation. However, little is known about the role of urban animals in transmission of both parasite species.

**Findings:**

A sensitive non-invasive genetic method was used to monitor *E. granulosus* and *E. multilocularis* infection among dog faecal samples collected from an urban area in Estonia in 2012–13. Out of 181 dog faecal samples analysed, 2.2% tested positive for *E. granulosus,* determined by sequencing as genotype G1. None of the samples tested positive for *E. multilocularis*.

**Conclusions:**

We report contamination of an urban environment with highly pathogenic *E. granulosus* G1 disseminated by dogs, and a potential risk to human health.

## Findings

### Background

Tapeworms of the genus *Echinococcus* are important parasites of mammals, causing life-threatening diseases called echinococcoses. Human echinococcoses are zoonotic diseases that in Europe are caused by two parasite species: *E. granulosus*, the causative agent of cystic echinococcosis or hydatid disease; and *E. multilocularis*, which causes alveolar echinococcosis [1]. In Estonia, as well as in the other Baltic States, the number of human cases of echinococcosis is increasing [2]. Human infections are most commonly associated with *E. granulosus sensu stricto* and, in particular, with its genotype G1 [3,4]. While *E. granulosus* s.s. in Europe mainly uses domestic dogs as definitive and domestic ungulates as intermediate hosts, the typical transmission cycle of *E. multilocularis* is wildlife-based, predominantly involving red foxes (*Vulpes vulpes*) as definitive and small rodents as intermediate hosts. Humans are considered as accidental intermediate hosts to both species, and they can be infected by ingesting parasite eggs via direct contact with a definitive host or through contact with contaminated water, soil or food. Due to their close contact with humans, dogs present a considerable risk factor in the spread of echinococcoses to humans [5].

Following recent increases in abundance, red foxes have started to occur regularly in European cities, including urban areas in Estonia [6]. Mirroring the range shift of its definitive host, *E. multilocularis* has also colonised the urban environment. The parasite has been recorded in foxes in a number of European cities [7,8] and recently also in Tartu, Estonia [9], prompting considerable concern for public health. To date the only report of *E. granulosus* infection in urban areas within the European Union occurred several decades ago when the parasite infection was detected in urban dogs in Rome [10]. However, the problem seems more serious than previously thought: in other European countries, including Montenegro and Serbia, the infection rate of urban dogs was found to be very high, up to 65% [11,12], and a cat with *E. granulosus* G1 cysts was recently found in Saint-Petersburg, Russian Federation [13].

The aim of this study was to monitor urban dog faecal samples for contamination with *E. granulosus* and *E. multilocularis* eggs using non-invasive genetics.

## Methods

### Non-invasive sample collection

**Figure 1:**
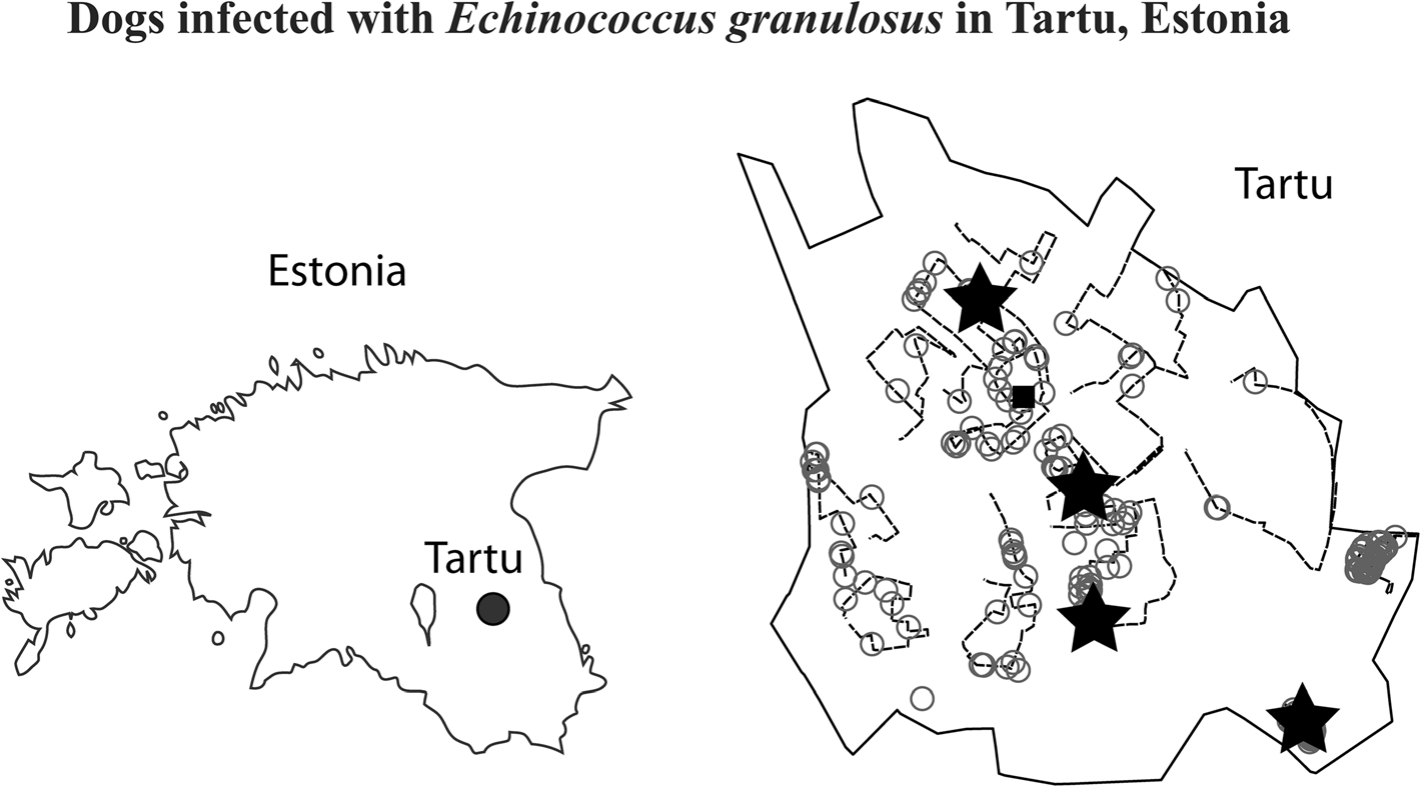
**The location of Tartu in Estonia (left), and the dog faeces survey area in Tartu (right).** Tartu city boundary is marked with a solid black line; survey transects are shown by dashed lines; and a rectangle marks the city centre of Tartu. Recorded dog faecal samples (181) are shown by open circles, while filled stars (4) indicate *E. granulosus* positive dog faecal samples.

Dog faecal samples were collected from January to March in 2012 and 2013 from the streets and green areas of Tartu, Estonia. We surveyed 14 transects, each approximately 4 km long (more than 850 km in total), incorporating all major districts in the city (Figure 1). Each transect was searched weekly during the study period. We collected 102 dog scat samples that were deep frozen at −80°C for at least one week to avoid the risk of *Echinococcus* infection [5], since both *E. multilocularis* [9,14] and *E. granulosus* [15,16] have recently been found in Estonia and have elsewhere been found to infect dogs [17-19].

### Molecular analysis

Samples were processed and PCR-based molecular analysis was carried out as described in Laurimaa et al. [9]. Essentially, scat samples of approximately 250 mg were placed into tubes, heated for 15 minutes and placed back at −80°C. The heating and cooling procedure helps to break the parasite egg shells, enabling efficient DNA extraction. DNA was extracted using the QIAamp DNA Stool Mini Kit (Qiagen) according to the manufacturer’s instructions. Primers F1/RVu and Dog1f/HW1r were used to amplify short sequences of mitochondrial DNA with total lengths of 76 bp and 56 bp to distinguish between fox and dog, respectively [9].

All samples were further analysed for *E. granulosus* and *E. multilocularis* using primer pairs specific to *E. multilocularis* (EMfor1, EMrev1) and *E. granulosus* (EGfor1, EGrev1), amplifying 120 bp and 149 bp sequences, respectively [9]. PCR was performed twice for each sample in a total volume of 20 μl using the touchdown protocol described in Laurimaa et al. [9]. All positive samples for *E. granulosus* were sequenced with the same primers as used for PCR. PCR product purification and sequencing procedures followed Saarma et al. [20].

## Results

**Figure 2:**
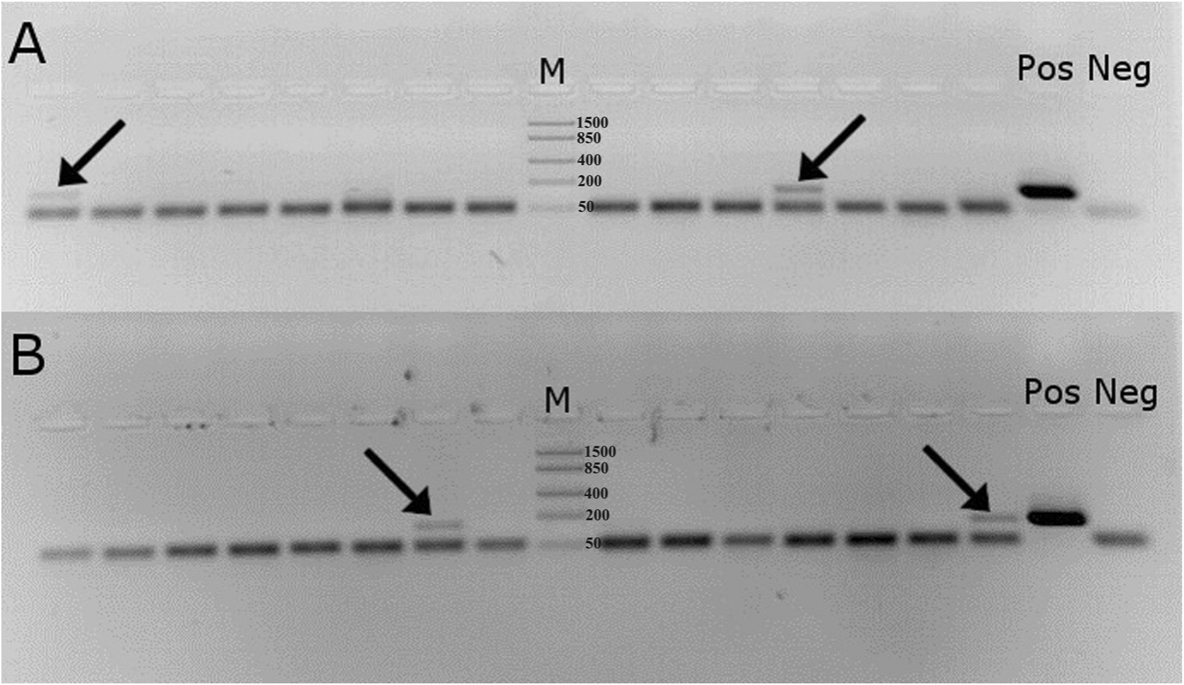
**PCR amplification of** ***Echinococcus granulosus*** **mitochondrial small-subunit rDNA fragment from dog faecal samples collected in Tartu (A and B).** Arrows point to the four PCR positive samples for *E. granulosus* (149 bp). Lane M: FastRuler Low Range DNA Ladder (Thermo Scientific; fragment sizes in base-pairs are as follows: 1500, 850, 400, 200 and 50); lane Neg: negative control; lane Pos: positive control.

DNA was successfully PCR-amplified from 90 samples out of 102 (success rate 88%). The PCR-based genetic analysis and sequencing verified that all samples belonged to dogs (data not shown). We also included 91 dog samples previously identified and analysed for *E. multilocularis* [9], but not for *E. granulosus*. Thus, in total a further 181 dog samples were analysed to determine infection with *E. granulosus*, and 90 samples were analysed for *E. multilocularis*. While none of the analysed dog samples were positive for *E. multilocularis*, we detected *E. granulosus* in four dog faecal samples (2.2%; 95% binomial confidence interval 0.6–5.6%; Figure 1; Figure 2).

**Figure 3:**
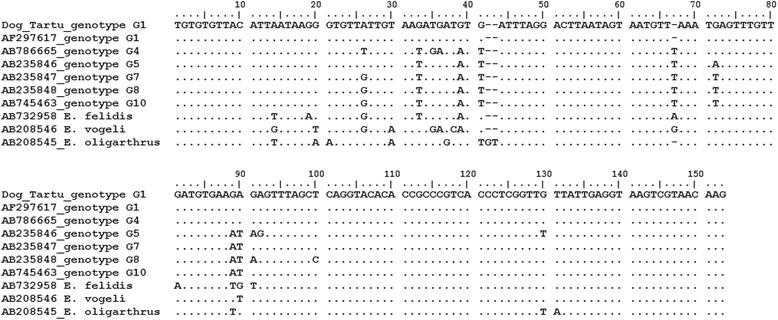
**Alignment of** ***E. granulosus*** **mitochondrial small-subunit rDNA sequences.** ’Dog_Tartu_genotype 1’ is the *E. granulosus* genotype G1 sequence obtained in this study, all others are homologous sequences from various *E. granulosus* genotypes and *Echinococcus* species from GenBank. The small-subunit rDNA fragment corresponds to positions 9984–10133 in AF297617. Note that the *E. granulosus* sequence obtained in this study shows 100% identity with the *E. granulosus* genotype G1 (AF297617). For other *Echinococcus* species the identity is lower (≤95%).

Sequence analysis demonstrated that *E. granulosus* found in urban dogs in Tartu belonged to genotype G1 (the ‘sheep strain’) and was 100% homologous to *E. granulosus* genotype 1 sequence AF297617 (pos. 9984–10133; small-subunit rDNA), whereas in comparison with the other *Echinococcus* species, the identity was ≤95%; Figure 3).

## Discussion

This study provides the first record of *E. granulosus* genotype G1 in dogs in a European urban environment. Moreover, while *E. multilocularis* has recently been reported in urban areas of the European Union, including Estonia [7-9], this is the first such record of *E. granulosus* for approximately 20 years. In rural areas of Estonia, both *E. multilocularis* and *E. granulosus sensu lato* are present: the former has been described in red foxes [14]; the latter in wolf (*Canis lupus*) and moose (*Alces alces*) [15,16]. Moks et al. [15,16] demonstrated that the sylvatic cycle of *E. granulosus* in Estonia involves moose (genotypes G8 and G10) as the primary intermediate host, and wolf (G10) as the definitive host. Roe deer (*Capreolus capreolus*) and wild boar (*Sus scrofa*) have also been examined for the presence of *E. granulosus*, and published results have reported no cysts [16]. Furthermore, *E. granulosus* has recently been reported in cows and pigs, but not in sheep or other farm animals, as reviewed in Marcinkute et al. [2].

Dogs usually become infected with *E. granulosus* genotype G1 after eating the discarded offal of wild or domestic ungulates [17,19]. Since feeding the viscera of wild and domesticated animals to dogs is commonly practiced, dogs may carry the parasite into urban environments following hunting trips in rural areas. Free-roaming stray dogs that move between rural and urban areas can also be a source of contamination. Since the genotype (G1) we detected differs from those recorded from wild mammals in Estonia (G8 and G10), an origin in the sylvatic cycle seems unlikely. The genotype G1 is most probably transmitted via the domestic cycle, and further sampling of production animals is necessary to determine the transmission path of *E. granulosus* G1 in more detail.

We expected that dogs included in this study might also harbour *E. multilocularis* infection, since about 30% of foxes in rural areas [14] and 7.1% of the analysed fox scats from the same urban area (Tartu) were found to be infected [9]. Moreover, 1.6% of raccoon dogs (*Nyctereutes procyonoides*) harboured the adult tapeworms in their small intestines (Laurimaa et al., unpublished). Given the relatively high densities of foxes and raccoon dogs in Estonia, both species seem to represent important definitive host species for *E. multilocularis* (and for other zoonotic pathogens in the country) [21]. However, none of the dogs analysed in Tartu were identified as *E. multilocularis*-positive, though such cases have been reported in Slovakia, where hunting rodents and feeding on raw viscera were found to be the main risk factors of *E. multilocularis* infection in dogs [17].

Decisions about the treatment and control of echinococcosis should rely on the accurate identification of *Echinococcus* species and genotypes. Morphological methods often fall short in such identifications due to specific limitations. Recent advances in the development of molecular methods have laid a solid basis for accurate detection of *Echinococcus* species and genotypes [9,22-26]. Nonetheless, it is highly desirable to have a sensitive and low-cost molecular diagnostic method that also allows *Echinococcus* parasites and their host species to be detected from degraded samples (e.g. faecal). The molecular diagnostics we used in Laurimaa et al. [9] and in this study is highly sensitive, being able to detect infection of a scat sample with a single *Echinococcus* egg. It is especially suitable for analyzing faecal samples since it uses relatively short sequences of mitochondrial DNA, allowing species-specific signals to be detected from samples where DNA may be highly degraded. As the method is based on regular PCR, it is also relatively low-cost.
